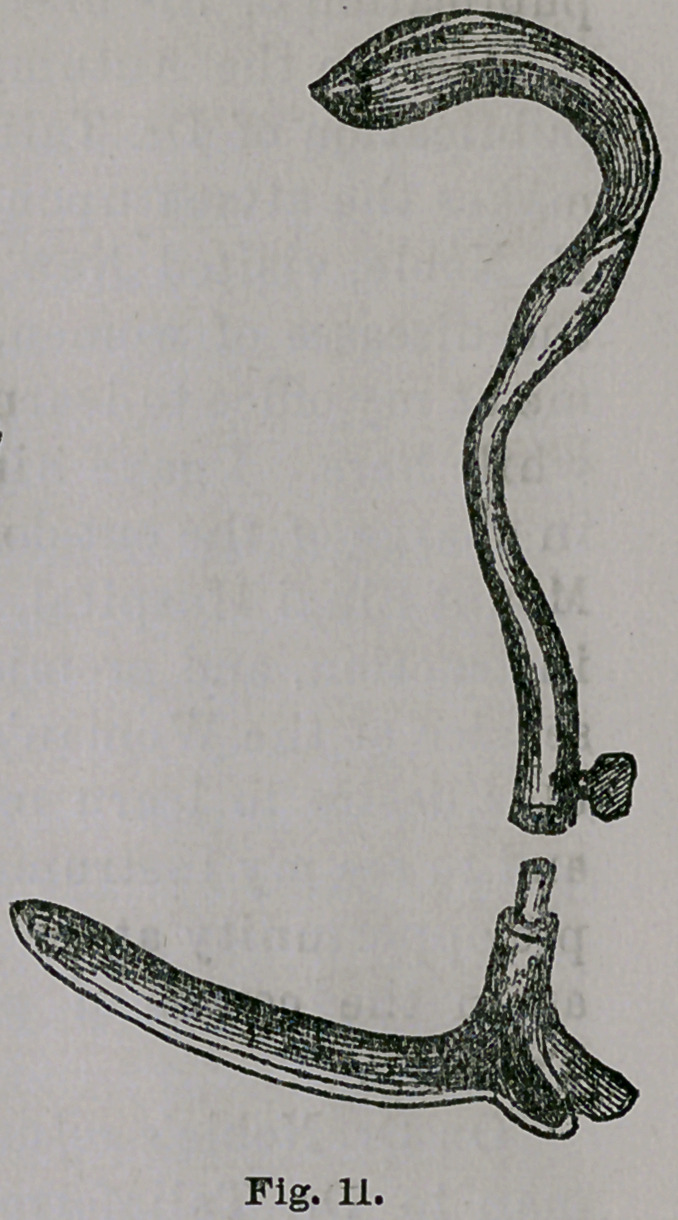# The Value of Graduated Pressure in the Treatment of Diseases of the Vagina, Uterus, Ovaries and Other Appendages

**Published:** 1883-01

**Authors:** Nathan Bozeman

**Affiliations:** New York, Surgeon of the Woman’s Hospital of the State of New York


ATLANTA

### MEDICAL REGISTER.

VOL. II.] JANUARY, 1883. [No. 4

### Original.

### THE VALUE OF GRADUATED PRESSURE IN THE TREATMENT OF DISEASES OF THE VAGINA, UTERUS, OVARIES AND OTHER APPENDAGES.

BY NATHAN BOZEMAN, M.D., NEW YORK,
Surgeon of the Woman's Hospital of the State of New York.

[Continued from Page 155.]

This brings us to the consideration of my paper presented to the American Gynæcological Society, September, 1878, to which reference has already been made at the beginning of this article. As it may not be generally understood, I will here repeat the objects I had in view in the preparation of that paper.

1. To record my protest against what I considered to be an abuse of the bloody operation for the superficial lacerations of the cervix uteri.

2. To explain what I believed to be the true "Mechanism of retroversion and prolapsus of the uterus considered in relation to the superficial lacerations of the cervix uteri."

3. To point out the relationship of prolapsus and imprisonment of the ovary, or ovaries, to the retro-displacements of the uterus, and especially to retroflexion with fixation.

4. To give a general idea of my mode of columning the vagina as a means of reducing to narrower limits bloody operations for superficial lacerations of the cervix uteri, and of successfully treating all forms of displacement of the uterus, ovaries and other appendages, including, also, pelvic exudations and adhesions.

5. To present to the notice of the profession a new vaginal support, constructed upon the principle of the parallelogram, and intended to supplement my method of columning the vagina.

In my remarks relating to the mechanism of prolapsus and retroversion of the uterus, I pointed out the counteracting forces concerned in maintaining the organ in its normal position; also endeavored to show that the displacements in question took place only when the expulsive forces of the body, residing mainly in the diaphragm and abdominal muscles, preponderated; that is, exceeded the pelvic forces by reason of the gradual development of the acquired forces, the result of disease. I further said there was not a single counteracting force residing in the cervix uteri below the vaginal attachment where superficial lacerations occur, and that any denudation and suturing of whatever notch, rent or laceration, occurring here from parturition, could not, from the very nature of things, however skillfully executed, elevate or restore the organ, as a whole, to its lost position any more than the simple denudation of the same parts and the processes of granulation and cicatrization could accomplish this result. The principle, I believed, had no foundation in logic nor in true surgery, and I still fail to see the importance attached to it by Dr. Emmet and other writers all over our country. To forcibly invert the lips of the cervix uteri for the purpose of elevating the whole organ, without mechanical support, is, to my mind, opposed to all the teachings of philosophy.

Laceration of the anterior lip of the cervix uteri with the vesico-vaginal septum will cause retroversion or retroflexion, and the reparation of both structures will cure the displacements. Why? Because one of the principal counteracting forces to hold the uterus in position resides in the vaginal walls, and to repair these lesions is to cure the particular form of displacement resulting from them. This, I believe, I was the first in this country to demonstrate. (See *New Orleans Medical Journal*, May, 1854, and *Southern Medical and Surgical Journal*, Augusta, Ga., August, 1855.)

In my paper under consideration, I spoke of complete procidentia uteri as a result of the yielding of all the counteracting forces, and said that the persistency alone of the integrity of the broad ligaments would prevent this form of displacement in the following manner: "While there is a joint action of the expulsive and the acquired forces, the broad ligaments may not yield at all, or only to a slight extent, and the further descent of the body of the uterus be prevented.

"In the event of the broad ligaments yielding, the same forces will drive the fundus farther down upon the rectum and thus cause retroflexion. *One or both ovaries often become involved in the process, are crowded against the rectum, and in that manner the most intense suffering is produced.*"

In my explanation of the mode of using dry cotton in the knee-elbow, or knee-chest position with a suitable speculum, (my self-retaining speculum) for the treatment of displacements of the *uterus and ovaries*, from whatever cause, I simply offered to the profession my own views upon this subject for what they were worth, and based them upon nearly twenty-five years experience with the use of sponge, hard rubber and cotton. This is shown as regards pressure with cotton in these words:

"There must be formed a firm pyramidal column of carbolized cotton or wool, extending from the posterior vaginal *cul-de-sac* obliquely downwards across the axis of the vagina to a point just within the pubic arch and the range of the perineum.

"By this mode of procedure it can be seen that, for the time being at least, the abdominal or expulsive forces are made to operate only at the greatest mechanical disadvantage; in other words, their action is reduced to the minimum degree.

"The uterus, the bladder and the rectum are thus made to gravitate beyond healthy limits, and so give the most perfect relief to all the structures in which the counteracting forces reside. The flattened column of cotton thus constructed with its base upwards, is in a position to support, not only the *uterus and the walls of the vagina, but also the ovaries, which are so frequently prolapsed in these cases.*

"The flattening of the column of cotton is intended also to save the rectum and bladder from undue pressure, such as may interfere with their functions. The narrowness of the column of cotton is also to be observed, in order that the lateral walls of the vagina may not be distended, but rather encouraged to contract.

"The pieces of cotton or wool with which the column is formed, may be secured in loops of strong sewing thread, so that the patient can remove them at the end of two or three days, and take the vaginal douche of warm water, preparatory to a renewal of the procedure. When the above indications are all fulfilled, the woman assumes the upright posture and performs her daily duties."

I also said that wool might be used, subject to the same rules, but certainly did not recommend it, for long before this time I had regarded it as far inferior to dry cotton, for the reasons already stated. With reference to the principle of columning the vagina and employing afterward a suitable vaginal support to complete the cure, I said:

"The *modus operandi* of this mechanical method of treatment, whether called *preparatory* or *curative*, is, I think, simple and can be easily understood by the patient.

"There are, however, objections to this plan of treatment:

"*First.* The time and attention, on the part of the physician, required to carry it out.

"*Second.* The prejudice of the physician against the knee-elbow position.

"*Third.* Defectiveness in the specula ordinarily employed, (including among them every form of univalve having simply a pubo-sacral or ante-posterior action.)

"*Fourth.* The pessaries in common use do not have the proper shape to give the required support after the preparatory treatment. * * * * *

"I have labored long to devise a suitable vaginal support to take the place of the carbolized cotton, but it is only within the past year I have succeeded in bringing the instrument to a degree of perfection which enables me to predict its ultimate success."

Of the general uses of this vaginal support, I further said:

"This instrument is not only useful for maintaining the uterus in an elevated position after retroversion and prolapsus have occurred, but it is also a most valuable instrument with which to accomplish the same end after the retroflexed and fixed uterus has been dislodged from the hollow of the sacrum by means of the cotton columns or compresses already described.

"After proper preparatory treatment, by means of the cotton columns directed obliquely against the vesico-vaginal septum from the perineum as *point d'appui*, the instrument can be used with equally satisfactory results in the cases of anteflexion and anteversion of the uterus. * *

"Suffice it to say that retroflexion and fixation of the uterus in the hollow of the sacrum, constitutes, both in the primiparæ and in the multiparæ, the largest class of uterine displacements, and often the most deplorable, which we are called upon to treat. Hitherto, treatment of these cases by means of the uterine sound and stem pessary has been unsatisfactory, and, according to my experience, a more comfortable, safe and effective method is unquestionably a great desideratum. The plan of treatment which I have described is nothing more nor less than an application of some of the principles of orthopedic surgery to uterine distortions, and I think will accomplish the end desired.

"Since I first adopted this plan of treatment, now, nearly seven years ago, I have relieved a number of most unpromising cases, such, I am sure, as could not have been cured by the use of the uterine sound and the stem pessary. These were cases in which one or both *ovaries were imprisoned and unduly compressed*, chiefly by the supplementing or acquired force residing in the rectum."

As to the principle and construction of my *vaginal support*, I will here quote the description then given:

"This instrument is constructed upon the principle of the parallelogram.

Fig. 5.

Fig. 6.

"It is elastic and thoroughly self-sustaining. The instrument is made of coiled steel wire. It has vesical and rectal branches which are covered with thin rubber up to points near the heel of the instrument, where an opening is left for the escape of the menstrual and other discharges.

"Upon the vesical branch is set a hair cushion which is to receive and support the vesico-vaginal septum. The covering of the rectal branch is distended with air in order that it may adapt itself uniformly to the recto-vaginal septum. The two upper uneven points are united by a broad elastic apron, which like a chair is to receive the cervix uteri, and to a certain extent to support the weight of the entire organ. When viewed edgewise, the instrument presents somewhat the appearance of a jockey's cap, and a medical friend suggested it should be called the "jockey cap" pessary. However, to avoid the name of a uterine pessary, I prefer to call it a *vaginal support.* This name is in strict accord with the action of the instrument, for it leaves the uterus and its relaxed ligaments to take care of themselves in their normal relation and position. This is an attainment of the highest aim I can conceive for any form of instrument employed for the latter purpose.

"This instrument is not only useful for maintaining the uterus in an elevated position after retroversion and prolapsus have occurred, but it is also a most valuable instrument with which to accomplish the same end after the retroflexed and fixed uterus has been dislodged from the hollow of the sacrum, by means of the cotton columns or compresses already described.

"After proper preparatory treatment, by means of the cotton columns directed obliquely against the vesico-vaginal septum from the perineum or *point d'appui*, the instrument can be used with equally satisfactory results in cases of anteflexion and anteversion of the uterus."

Fig. 7.

After the foregoing description of this vaginal support was published, I made some important improvements in its construction. These improvements will be readily appreciated by reference to Fig. 6; first, the hair cuhsion on the vesical, or upper branch, is replaced by plain and solid rubber; second, the air-distended covering of the rectal branch is now simply the double thickness of the rubber; and third, the elastic apron extending only around the points of the two branches is now carried from the toe toward the heel of the instrument about two-thirds the distance. Beside these noteworthy changes in the general configuration of the instrument, it is now much lighter, thus admitting of greater elasticity between the sides and branches. The extension of the elastic apron proved to be of inestimable value in preventing the lateral walls of the vagina from falling between the vesical and rectal branches. Possible injury to the cervix uteri from the edge of the old apron is also obviated.

Fig. 7, in section, illustrates the instrument in position, both as regards ante and retro-flexions with prolapse of the ovary (*o*) in Douglas' fossa. The positions (*g* and *b*) indicate the points of supposed displacement of the uterus, and the reversal of the arrows (*e* and *a*), the general direction of the two forces by which the organ from anterior or posterior columning of the vagina, has been carried to its present normal position. The divisions of the two branches at their centre are shown (at 1 and 2) with the elastic apron stretching from one point to another and supporting the uterus as in a swing. The arrows (*e* and *a*) represent the anterior and posterior lines of *descensu uteri* under the operation of pressure and the expulsive forces, residing in the diaphragm and abdominal muscles, supplemented by the acquired forces of disease. The first line of descent in unopposed anteflexion of the uterus, would be towards the star at the junction of the perineum with the recto vaginal septum; the second in retroflexion towards the star on vesico-vaginal septum opposite the root of the urethra. Any form of pessary, therefore, with a single branch, acting in either of these lines, intended for anteflexion or retroflexion, must necessarily find a centre at one or the other of these stars, with the resulting impingement or pressure upon the opposing hollow organs. Unite, however, the two single branches in accordance with the principle of the parallelogram and the effect of the resultant will be found at the circled star indicated by the arrow (*c*). As the acquivalent of all the component forces arising from downward movement or pressure of the uterus and walls of the vagina.

(*"By any number of forces acting together for a given time, a body is brought to the same place as if each of the forces, or one equal and parallel to it, had acted on the body separately and successively for an equal time."—Silliman.*)

Thus is placed in the vagina an instrument which is elastic in every direction upon which pressure falls, self-acting and self-sustaining in the axis of the organ, with no tendency to escape when properly adjusted. By the side action at the heel of the instrument, the same as that of the antero-posterior, it is rendered additionally secure against the several forces directed upon it from above, before and behind. In the adjustment for anteflexion of the uterus, the cervix is brought from behind the extremity of the rectal branch (2) and made to stand on the elastic apron, while the extremity of the vesical branch (1) aids in preventing the falling forward of the body of the organ upon the bladder; for retroflexion, the cervix uteri is caught upon the elastic apron beneath the extremity of the vesical arch (1) and carried back to the same position as in anteflexion, as is here represented in the cut. Thus, in both forms of displacement, it is seen that the cervix uteri—the short arm of the lever—is acted upon instead of the long arm, as is the case in the use of the Hodge's pessary and its various modifications.

The advantages of the mechanism of my instrument over the latter, with regard to the ovary (*o*) in the retro-peritoneal fossa, are self-evident, not only as seen in this diagram, but as actually observed in my every-day practice.

Before leaving this part of my subject, it is proper for me to say that any one employing this instrument with the idea that he can introduce it into the vagina and relieve any sort of uterine and ovarian displacement, without the necessary preparatory treatment, by vaginal columning, will soon find himself doomed to sad disappointment.

The successful use of the instrument depends upon the thoroughness with which vaginal distortions and uterine displacements are first overcome by the columning of the vagina, supplemented or not, according to circumstances, by the pressure of sponges in bags of oil-silk or *taffetas de soie.* The great value of the instrument is to maintain the cure and to enable the patient herself to do this, especially in the large class of cases where a hard pessary cannot be borne.

It is true there are many cases in which there is retroflexion of the uterus with fixation, and in which it is impossible to elevate the organ beyond the axis of the vagina. Even in such cases, the instrument will hold the uterus, with the ovary when prolapsed, up to this line with the greatest comfort to the patient, and thus enable her to lead a life of moderate activity, which she could not do with a hard instrument requiring constant watching by a physician.

This instrument is intended to be worn only during the day, when the patient is upon her feet. She removes it at night and introduces it in the morning. The greatest objection I have heard urged against the use of the instrument is the disagreeableness of the odor produced by the warmth of the body and the ordinary secretions of the vagina and uterus, but this is an objection generally urged by physicians and not by patients. The latter are usually more impressed by the benefits derived from wearing it than by any unpleasant odor. In fact, when the patient can afford it, and two instruments are at hand for alternate use, the objection indicated may be almost entirely overcome. A solution of permanganate of potash (℈j--℥i), in which the instrument may be left over night, contributes greatly to cleanliness.

There is another fact I will state in regard to this instrument, which is necessary to be known in order to appreciate the limit of its application; it is that a higher degree of intelligence is required for its use than is to be found in the lower walks of life. In short, the patient must have sufficient intelligence to understand the character of the displacement under which she labors, and the object sought to be attained by the instrument placed in her hands for the maintenance of the cure. On these points, however, I find no trouble in private practice. In fact, I sometimes have patients so familiar with the use of the instrument, and so thoroughly relieved from their troubles of years' standing, that they will not lay it aside when they are really cured, fearing a relapse. I could cite a number of cases of this class if I had the space. One from the State of Georgia I may be permitted to mention. This patient had retroflexion of the uterus with fixation and imprisonment of one ovary, and was on the verge of insanity when she consulted me in the autumn of 1879. From posterior columning of the vagina she soon recovered her health, and gained upwards of twenty pounds in weight in less than a year. She has now been wearing the instrument nearly three years, and says it is no more trouble to her than putting on her stocking, and she means to wear it as long as she lives. Another case is that of a young unmarried lady of New York who came to me for treatment May 17th, 1878. She suffered from a bad form of anteflexion of the uterus. She was treated by anterior columning of the vagina and cured. My vaginal support was introduced to maintain the cure, and soon afterwards she left for Europe with her parents for a two years' trip. Notwithstanding she was told to lay aside the instrument after three or four months, she continued to wear it the whole time she was abroad, having fresh instruments sent regularly from New York every five or six months. On her return home she reported herself at my office, well and hearty, with a rosy complexion, saying the support was no trouble to her, and that she preferred to wear it longer, rather than take any risk of a relapse. Finding her thoroughly cured in every particular, I again advised her to stop the use of the instrument.

I will mention another fact in relation to treatment by columning the vagina and employment of the vaginal support, that is, restoration of the lost power on the part of the patient to exercise in the open air and to maintain her wonted interest in out-door life. The uninterrupted monotony of home life, and the varying scenes of hospital residence, sometimes gloomy and depressing, are not always conducive to mental and physical effort, and something is needed to arouse her dormant energies and restore confidence, that she may be able to perform those daily duties which were formerly a pleasure to her. That something is the elèvation and support of the uterus and its appendages to the highest limit of their tether, and relief, not only from actual pain, but from that "dragging, good-for nothing feeling," with cold hands and heavy lower extremities which only such patients know how to describe. Once prove to such a patient the practicability of accomplishing this, confidence comes and often enthusiasm, at the prospect of regaining her lost health and the power of well-doing. By far the largest proportion of cases in my private practice belong to this class. After having been under the care of a dozen or more physicians, and in not a few instances having passed through the trying ordeal of a bloody operation for laceration of the cervix uteri alone, or in conjunction with a bloody operation upon the anterior vaginal wall, or in conjunction with both of these an operation upon the perineum. In these very cases, wholly unrelieved by these successive bloody operations, where the perineum had even to be cut down to its normal depth to allow treatment, I have succeeded in an incredibly short time in restoring confidence by columning the vagina alone, and securing power to exercise in the open air. I have even had patients, knowing the muscular and nervous strain of a day's visiting or shopping, who have come to me for treatment in the morning before beginning their round, saying it was only by this aid that they could hope to carry out their intentions. With my vaginal support, under such circumstances, the same power to undergo fatigue is maintained, and a cure ultimately effected by the individual efforts, as it were, of the patient herself.

From the general character of my paper in 1878, following upon my long experience in the treatment of diseases of the vagina and the uterus by graduated pressure, I think it will be conceded that I had at least decided views at that time upon the several points here brought out, and perhaps none more positive than those with reference to the bloody operation for superficial lacerations of the cervix uteri, and also the frequent relationship of prolapsed ovaries to retroversion and retroflexion of the uterus. So, also, with regard to columning the vagina which I had already been using for years in connection with my intra-ischial or bilateral acting speculum.

In September, 1879, at the annual meeting of the American Gynæcological Society, held in Baltimore, Dr. P. F. Munde, of New York, submitted a paper on "Prolapse of the Ovaries," and in the discussion which followed, in which several of the members took part and corroborated with marked unanimity his statement with regard to the value of the "cotton tampon" in the treatment, I said:

"According to my observation, prolapse of the ovaries is of frequent occurrence, and it is a subject which has interested me for many years. I agree fully with Dr. Skene and Dr. Goodell with reference to the mechanism by which the symptoms are produced. I am not sure, however, but that, in the majority of cases, it is the retroflexion, which is almost invariably associated with the prolapse, that gives rise to the pain, rather than the difficulty in defacation. I have seen cases in which there was almost complete obstruction of the bowel, in which not only was pain occasioned by the retention of the feces, but the habit of constipation was the result.

"With reference to disease of the organ, it certainly exists in many cases, although, as Dr. Munde has stated, it is not, to all appearance, diseased in many cases. I recall one case in which the ovary was prolapsed into Douglas' pouch, where it became fixed to the uterus, and underwent cystic degeneration, rupturing and discharging into the uterus at two different times.

"The treatment which has been proposed by the author of the paper is very good, so far as it applies to simple cases. But where the organ is prolapsed, and has become fixed, I think he has not given sufficient importance to pressure while the patient is in the knee-elbow position; cylinders of cotton are placed in the vagina, so as to bring pressure directly against the fixed ovary.

"I have found the iodoform ointment, applied to the *cul-de sac* and kept in place by a column of carbolized cotton, of great value to relieve the tenderness and hyperesthesia which always exists in these cases. As Dr. Munde has mentioned my name in connection with support of the prolapsed organ by means of the cotton vaginal tampon, I will merely say that such has been my practice for the last twenty years.

"I have seen fixation of the ovary not only by retroflexion and latero-flexion of the uterus, but it is also often fixed in the posterior *cul-de-sac* low down, by anteversion, thus giving rise to the same symptoms that have been mentioned at considerable length. I have seen the greatest relief of the symptoms from the use of pressure when the ovary is prolapsed and adherent. Of course if the ovary is firmly fixed in that position it is not to be supposed that by pressure it can be disengaged, but it is possible to carry the uterus up and with it the ovary, by which means the vesical symptoms are relieved."

In September, 1881, at the annual meeting of the same society, in New York, Dr. Ely Van De Warker read a paper entitled "Forcible Elongation of Pelvic Adhesions," which was also discussed by a large number of the members, and with singular unanimity the practice was condemned. For my part, I said:

"I have been much interested in this class of cases, retroflexion with fixation of the uterus, frequently accompanied by prolapse of the ovary. My attention was first called to it in 1859, in connection with a vesico-utero-vaginal fistule. My operation, at that time, was to make incisions from the right and left angles of the fistule through the vesico-vaginal septum, for the purpose of disengaging the uterus, and then to place cylinders of sponge in oiled silk bags in the vagina. These cylinders were introduced and crowded into the posterior *cul-de-sac* daily, and the treatment was kept up for weeks. I found that it was possible by this means to restore the uterus ultimately to its proper position and thus disengage the cervix from the bladder. I have also treated these cases complicated with incarceration of the cervix in the bladder with carbolized cotton, "columning the vagina," and this method I brought before this Society in 1878 and 1879. It is a practice to which I resort almost daily, and consists simply in placing the patient in the knee-elbow position, and making these columns in the vagina rest against the pubic arch and the perineum. By this continuous pressure I have been able to stretch the posterior vaginal wall of not more than two and one-half inches in length, to the depth of five or six inches, thus gradually loosening the uterus, so that it has been restored to its natural position. These columns are usually allowed to remain about thirty-six hours, when they are removed by the patient by means of the little cords attached, and the vaginal douche of warm water is used. At the end of twelve or twenty-four hours they may be renewed. Of course other means may be employed in connection with this method, as iodine, hot water, etc. The principle of elongating the posterior wall of the vagina is most important. When the elongation has been completed, and the uterus brought into proper position, the ovary is usually restored with it, and the patient can, as a rule, wear a Hodge's pessary or my vaginal support, presented to the Society in 1878"

From these remarks it will be seen that I had already given special attention, when these two papers were discussed, not only to the pathology and mechanism of prolapsed ovaries and pelvic adhesions, but to their successful treatment. I have introduced the above report to show the progress and development of what I have come to regard as the only rational method of dealing with such difficulties.

We will next turn our attention to the published views of one or two other physicians who have had experience with my mode of columning the vagina in diseases of the uterus and ovaries.

Dr. Rudolph Tanszky, of New York, gynæcologist to the Out-door Department of Mt. Sinai Hospital, whose attention was directed to my special modes of treating intra-vaginal and utero-ovarian diseases as early as 1872, mentions sixteen cases of prolapsus of the ovary and fifty-eight cases of retroversion and retroflexion of the uterus in 371 patients applying for treatment from December 1st, 1877, to December 1st, 1878. The mention of these cases can be found in the report of the Hospital for that year, and he treated them by columning the vagina according to my method.

Here is a proportion, then, of 27.58 per cent. of cases of prolapsed ovaries out of 58 cases of posterior displacements, and 4.30 per cent. out of 371, the whole number of cases treated. This statement is interesting from a statistical point of view.

Dr. Tanszky's estimate of the value of my method of columning the vagina, after several years experience with it, may be inferred from an article by him in *The New York Hospital Gazette*, April 5, 1879, entitled "The Tamponade of the Vagina Successfully Applied as a Curative

Agent for Uterine Displacements with Adhesions and Prolapsed Ovary." He ably discusses the pathology of displacements of the uterus and ovaries and their treatment, and especially of chronic inflammation of the womb when retroverted instead of retroflexed, giving the prevailing views of leading writers upon this latter point as well as upon the proposal of the bloody operation, or trachelorrhaphy, as it is now sometimes called, for superficial laceration of the cervix uteri when this lesion is found to exist. After acknowledging the failure of all the ordinary resources, including trachelorrhaphy, to relieve the cases of retroversion of the uterus with adhesions, he proceeds to give his experience with my vaginal columns of cotton as follows:

"But still there were the adhesions and the displacement which no surgical operation, no internal or external support of the womb, and no medication known to me, would have relieved to such a satisfactory degree as the plan for accomplishing this purpose first used, I am told, twenty years ago by Dr. Nathan Bozeman, of this city, and which I have myself found to be of the highest value in the treatment of complicated or uncomplicated cases of uterine adhesions and displacements of the ovary in private practice as well as also in my service in the Mt. Sinai Hospital Out-door Department. This method consists in the gradual stretching, elongation of the vagina by means of carbolized cotton, (the use of carbolized cotton, of course, instead of the ordinary cotton is of recent date).

"The simplicity, the safety, and the usefulness of the method for which the profession is indebted to Dr. Bozeman who claims, and with justice, the priority of this mode of treating uterine displacements and adhesions whether complicated with *ovarian prolapse* or not, will be apparent to the most skeptical after trial. * * * *

"The *rationale* of Bozeman's method of tamponing the vagina for the relief of uterine adhesions seems to me to be the following: The vagina is elongated and put somewhat upon a gentle stretch; the rugæ become smoothed out; the fornix vaginæ is elevated in the pelvis; the adherent uterus, ovary, etc., are supported from below upwards by the soft cushion thus applied; the blood-vessels are relieved from distension and their hyperæmic state, the plexuses and nerve filaments are also thereby relieved from direct pressure from the enlarged, fixed and displaced womb, and the surrounding, often accompanying exudation, which, if within the ligaments, may be gently and gradually moved. The cautiously exerted pressure, through the column of the cotton in the vagina, acts as a stimulus to the lymphatics and promotes absorption of first liquefied peri-uterine exudations. The bladder also being supported by the tampon, is more readily emptied than before, and often the great distress of painful and frequent micturition is greatly lessened. It is hardly necessary to state that each tampon has a string attached to it, for the purpose of its easier removal. The tampon remains for forty-eight hours usually, when the vaginal douche is used and the tampon is re-applied. In a few weeks the good results are manifest by the more comfortable feelings of the patient and the mobility of the uterus found to exist by the examining surgeon.

"Since uterine adhesions and chronic pelvic exudations have heretofore constituted a large majority of incurable cases in gynæcological practice, the attention of the profession is hereby called to a simple method of relief, which it has proved to be in my hands at least, and those of Dr. Nathan Bozeman, to whose kindness I am indebted for having first called my attention to it."

In February, 1882, at the annual meeting of the New York State Medical Society at Albany, Dr. William Warren Potter, of Buffalo, submitted a paper entitled "The Genu-Pectoral Posture in the Treatment of Retro-Displacements of the Uterus, and in Dislocation of the Ovaries," in which he spoke of this position (meaning the exaggerated knee-elbow or knee-head position), of my speculum and of my mode of columning the vagina, as follows:

"Having accustomed myself, during several years past, to employ the knee-chest position in the treatment of all backward displacements of the uterus, as well as in prolapse of the ovaries, I am prepared to affirm the superiority of this method over all others with which I am familiar, in the management of this class of maladies. Under its timely and judicious use, even the most complicated and obstinate kinds of retroversion and retroflexion, with fixation of the uterus in the hollow of the sacrum, may be made to yield. * * * *

"Of the speculum: It is important, for knee-chest uses, that the speculum should be constructed with laterally expanding blades, and be more or less self-retaining. A perineal elevator with a convenient handle, and a flattened blade, goes to make up an essential part of the instrumental equipment. These requirements are admirably met in the instruments devised by Dr. Bozeman, and which bear his name. While the Sims' speculum is, undoubtedly, possessed of a wider range of usefulness than any other speculum, and may be made vastly serviceable in the genu-pectoral posture, yet for purely knee-chest purposes the Bozeman instruments are superior to any with which I am familiar. * * * *

"In every case it is my custom to commence the treatment by filling the post-cervical space of the vagina with the pledgets of cotton already described. The first two pieces are usually saturated with carbolated glycerine (one per cent. carbolic acid) and placed well behind the cervix, covering the os uteri, to be quickly followed with other dry bits, until a column is built down to the pubic arch. This is done through the Bozeman speculum, and each pledget, as it leaves the forceps, is caught by the distal end of the perineal lever, and gently but firmly carried to its place. The lever is first withdrawn, then the speculum, and finally the right index finger is introduced to steady the cotton column, while the patient is resuming the erect posture."

Although Dr. P. does not acknowledge, in his description of the process of introducing the pledgets of cotton into the vagina through my speculum, the source of his information, still it is precisely given as he learned it in my office and in my service at the Woman's Hospital.

Thus far in my historical sketch of the value of graduated pressure in the treatment of diseases of the vagina, uterus, ovaries and other appendages, I have confined myself strictly to the statement of facts, avoiding controversies entirely. I wish I could now feel that it was unnecessary to say anything as to the bearings of the above recognized advances in gynæcological surgery, or in vindication of my claims to originality. I shall add, however, only what seems to be required to refute unjust criticism.

I may, therefore, be pardoned for again referring to Dr. Campbell's paper, published in 1876. (Op. cit.) On page 231, after pointing out the advantages of the exaggerated knee-elbow, or, technically speaking, the *genu-cephalic* position, in a simple case of retroversion of the uterus, where "reversal of gravity," "draft of the viscera," and "pneumatic pressure" having in his hands reached their highest point of effectiveness, he remarks, "No *dilating* speculum is now required," (italics his), referring, no doubt, to my intra-ischial or bilateral-acting speculum. I say my speculum, because I know of no other instrument capable of such dilatation of the vagina as he describes, not only in such passive cases, but, when necessary, independently of the will and resistance of the patient, and it is hardly probable that he knew of any other such instrument at that time. Having avoided in his paper making mention of, or alluding in the slightest way to, my designating term "knee-chest" for the double right angle triangle position of the body of the patient upon my supporting and confining apparatus, and having then used the same term under the Latin guise of "*genu-pectoral*" for the old exaggerated knee-elbow position, as shown in his cut, Fig. 2, presented on a former page, without acknowledging indebtedness, the inference, I think, is legitimate that his intention was also to ignore my speculum; hence the special allusion to the latter, as shown by his italicizing the usual qualifying word "*dilating*." It is no doubt true, that "no *dilating* speculum" was needed to produce the results indicated by placing his patient upon her head, or as near it as he could, in the genu-cephalic position, as shown by his cut.

Having, as he did, the full co-operation of the patient and no adhesions of the uterus, gravitation of the abdominal viscera with the pelvic was all he really needed for his replacement. The uterus being naturally drawn into the vacuum thus made righted itself which would have been impossible in the large class of cases where co-operation of the patient is *nil* and adhesions exist for which my "dilating speculum" is especially adapted, as was shown in my typical case cited in connection with the first use of this form of speculum, November 20th, 1867, and from which Dr. C. doubtless first learned the emphasized peculiarity indicated. In this case there was anteversion of the uterus and an abnormally large vagina with relaxed walls, and associated with these conditions there was a small vesico-vaginal fistule high up having cicatricial and plated borders which it was important to display for operative purposes. The patient being incapable in the knee-head or any other position of giving her co-operation even with all the univalve specula and assistants that could be pressed into service, besides having fifteen pounds of atmospheric pressure to the square inch to aid her, she could not afford a complete view of her little fistule so much concealed. Here "reversal of gravity," "draft of the viscera" and "automatic reduction by pneumatic pressure" had *full sway*, and yet after their utter failure, this woman had to be placed finally upon my supporting and confining apparatus in the *knee-chest* position, under the influence of an anæsthetic, in order to show this seemingly insignificant fistule. This was done, independently of her will and resistance with this same "dilating speculum," and the fistule was closed in less than twenty-five minutes, without assistants further than to administer the anæsthetic and to hand sponges. (See *N. Y. Medical Record*, January 1, 1868).

I do not find fault with Dr. Campbell for omitting to mention all this in his paper, but I do complain of his misuse of my designating term *knee-chest position* for his *knee-head position.* In scientific progress and honorable rivalry we all like to be in accord in thought and well-doing. If I have not properly utilized and named this knee-chest position in connection with my supporting and confining apparatus, or even my "dilating speculum," it remains for Dr. C. to inform the profession at large in what particulars I have failed to secure their legitimate results in practice.

There are other uses to which the knee-chest position has been applied in connection with my supporting and confining apparatus, and for which my designating term has been likewise misused. An important one is that of vaginal ovaritomy by Dr. T. G. Thomas. In the fifth edition of his work on "Diseases of Women," page 731, he illustrates the position with the usual cut as "Bozeman's Securing Apparatus," giving at the same time the following description: "The patient having been etherized was placed in the *knee-elbow position* (italics mine), and secured upon the apparatus of Dr. Bozeman. This apparatus not only completely fixes the patient in the position by straps and braces, but makes the position perfectly comfortable for any length of time, and also favors the administration of an anæsthetic."

How Dr. T., with his usual accuracy in speaking and writing, could so overlook the laws of mechanics and physiology as to make this mistake of calling the knee-chest the knee-elbow position I do not understand, especially after he has pointed out so lucidly the advantages of the position as to comfort and security of the patient which he knew could be obtained in no other anterior position than the *knee-chest.*

We come now to an examination of Dr. Taliaferro's claims to notice to which reference was made at the outset of these remarks. For this purpose it is only necessary, I conceive, to show what he did publish in the spring of 1878, with regard to pressure with sheep's wool in diseases of the uterus; what changes he has since made in the principle of packing the vagina, and how he was led first to adopt the practice. (Reprint from the Transactions of the Medical Association of Georgia.) But before proceeding further it is proper to state that Dr. T., in his practice, adopts the exaggerated knee-elbow or genu-cephalic position of the patient, and uses the pubo-sacral or antero-posterior dilatation of the vagina with the univalve or Sims' speculum.

First. *Pressure with sheep's wool in diseases of the uterus* The mode of introducing the wool is as follows: "The pledgets of wool are then successively applied, dry, each one being first rolled upon itself rather tightly in order to give the requisite firmness and solidity to the packing. The vault of the vagina is first well filled and the packing proceeded with *carefully*, the pledgets rolled upon themselves, being placed here and there, and *packed* with probe or dressing forceps; all parts of the vagina being packed as equally firm as possible, and yet not too solid at any point for discomfort. The vaginal canal is thus filled down to the muscular floor of the pelvis, but not below it." (Italics his.)

Fig 8.

Fig. 8, copied and introduced here, being about one-fourth size, shows the limit of pubo-sacral dilatation of the vagina with Sims' univalve speculum in position, and the space occupied by the wool, incident to such action of this form of speculum. The vagina is seen to be packed from the vault to the floor, and crowded backward and forward almost to the pelvic bones. The measurement, antero-posteriorly, on the basis of the size indicated, is three inches, about half an inch less than the outlet of the pelvis or the coccy-pubic diameter. As to the proportion of space allotted to the urethra and rectum respectively, for the performance of their physiological functions, it is not stated, but we are told that the tampon, so introduced, is to remain two or three days, when it is removed, with forceps, by the physician.

A glance at the construction of this irregular and somewhat quadrilateral-shaped body, almost solid, leads one to conclude, that the model for its construction was the fœtal head, occupying the pelvic cavity, and having a similar mechanism with the antero-posterior diameter corresponding to the occipito-frontal. As the uterus is the favored organ of the pelvis, being placed above the range of antero-posterior pressure and having a shelf upon which to rest, it is out of harm's way, if not of disease, certainly, of the innovations of art. Whether any woman is capable of enduring such mechanical pressure upon her rectum and bladder the two or three days it is allowed to continue is an open question, but it is not necessary here to discuss this point further. Any one who has had experience in tamponing the vagina for the purpose of controlling uterine hemorrhage can form his own opinion as to the principle underlying the practice, the value claimed for it, and the suffering, if not the actual danger attending the mode of use.

Second. *Change in the principle of packing the vagina.* Four years later, (*Atlanta Medical Register*, September 1882), Dr. T. furnishes us with an account of his improved mode of instituting pressure with cotton in diseases of the uterus, including also, this time, the ovaries. This is his mode of introducing the cotton :

"I have long since discarded the sheep's wool for cotton, which I originally used. I was induced to do this because of the superior convenience of the cotton, and because it can be made more compact, and hence a greater degree of pressure obtained. Instead of extending the tampon from the vault of the vagina to its floor, I now rarely extend it further than the upper third of the vagina, and often not more than the upper fourth. In case an extra degree of pressure is desired, the upper half or still more rarely the upper two-thirds of the vagina is packed. In the large majority of cases of congestion, displacements and adhesions, the tampon is made to occupy only the upper fourth of the vagina. If tenderness of the organs admits it, this partial tampon should be *very firm.* If there is considerable tenderness the dressing should be very light, and the pressure gradually increased as the tenderness subsides, until the packing is made as firm and compact as it can be made. When *pressure* is desired, a loose packing is not sufficient, and the so-called columns of Bozeman are worthless. This column is simply a loose, flat tampon extending from the posterior *cul-de-sac* to the ostium vaginæ. It can neither give *support* or *pressure* to the uterine organs. Its value consists mainly in separating and possibly softening the vaginal walls, for which it was used by its author until he read my paper in the spring of 1878.

"The tampon applied only to the upper third of the vagina does not interfere with the bladder or rectum by its pressure upon these organs. The lower portion of the vagina closes in the tampon which occupies its vault. The tampon is thus securely held in place and rests upon the elastic column formed by the approximated vaginal walls.

"The advantages of the tampon thus applied are: 1. It does not reach the urethra nor make uncomfortable pressure upon the bladder and rectum. 2. It answers all the

Fig. 9.

purposes of pressure of the more extensive tampon. 3. It does not interrupt the physiological mobility of the uterus. 4. It keeps in place more securely than the more extensive tampon.

"In witnessing the application of the tampon by my medical friends, some of whom are familiar with uterine manipulations, I have been astounded at the *awkward* manner in which the operation is made.

"Almost invariably, such a tampon utterly fails in its objects, and is worn by the patient with great discomfort. The packing should always be light and firm in the vaginal vault, and if this is properly done, no advantage is gained by filling the lower part of the vagina." (Italics his).

I introduce here the accompanying illustration, Fig. 9, which I have copied partly in section only, and lettered it with arrows to enable me to explain the principle. I do not hold myself responsible for the anatomical inaccuracies displayed in the cut; I simply represent in my copy the vagina, rectum, bladder and uterus, and their relationship to the pelvic bones as they are delineated in the original cut.

Compare this illustration with the preceding, Fig. 8, and it will be seen to differ in several very essential particulars. The vagina is here represented as being occupied only in its upper half with a flattened column extending from above obliquely downward and forward.

The rectum and bladder, instead of being crowded apart to the limit of a child's head, as indicated by Fig. 8, are here represented in the lower half of the vagina as being almost in contact. In short, the rectum is relieved entirely, and the bladder partially from pressure, due to diminution in the size and form of the column. The points 1 and 2 show the limit of former packing and distension of the vagina.

The flattened column, instead of acting antero-posteriorly, as the wool in bulk formerly did, is made to act longitudinally, somewhat parallel, with the axis of the vagina, with one end extending loosely up into the posterior *cul-de-sac* and the other resting upon the upper part of the vesico-vaginal septum as *point d'appui.* The bladder, instead of being empty and compressed against the pubis by the wool, packed as formerly, is here represented distended and elongated from the horizontal dotted line (*a*) obliquely across the axis of the vagina to form a cushion between the pubis and the lower end of the column. This displays a peculiar mechanical ingenuity, because it illustrates an attempt to utilize a physiologically acting, hollow organ to counteract the law of gravitation.

The uterus, instead of standing in a somewhat natural position or being shelved, so to speak, upon the hard-packed wool tampon above the line of pubo-sacral pressure, is here represented in a partially anteverted position, supposed to have been elevated to that point by the flattened short column, from some unknown retroverted position below the horizontal dotted line (*c*). The organ being thus elevated and sustained, in opposition to a well-known law in mechanics, to the effect that when two parallel forces act in opposite directions, the result is a revolution around the center, as at the cervix uteri, it naturally falls backward to its original position. The operation of this law, therefore, in the illustration before us, the uterus being on the line of the arrow (*b*), is to carry the short, flattened and somewhat semi-lunar shaped column to the point (1) in the direction of the arrow (*d*), or as far that way as the distended and already overworked bladder will permit. Such a column of glycerated cotton loosely lodged, as here shown, in the posterior vaginal *cul-de-sac,* without natural support below, and acting somewhat like a marble under the tongue, is wholly insufficient to give the uterus the support it requires in the position indicated. And as to the practicability of making the distended bladder, as shown, or rather the upper part of the vesico-vaginal septum, hold up the column, the proposition is simply an absurdity.

The question may be asked, in what other way could this uterus be prevented from falling backward and causing this form of column to revolve as around a centre, as above shown? I answer, simply by taking the star (*a*) opposite the root of the urethra in its normal relation as a *point d'appui* for the construction of the required column.

Supposing the fundus of the uterus, whether retroverted or retroflexed, to be at the point (2) as indicated in the same cut, the direction of the force by graduated pressure to elevate the organ would be at first to this point from star (*a*). This force, apportioned of course to the resistance to be overcome and the sensibility of the parts, gradually elevates the uterus and its appendages to the horizontal dotted line (*c*), when both stars, (*a* and *e*), the opposing walls of the vagina, become in common the *point d'appui*. The column is now parallel with the axis of the vagina, but the pressure being kept up, the *point d'appui* is, by degrees, changed to star (*e*) and the line of force to arrow (*c*). Thus is given to the uterus the support necessary to maintain it in the upright position, and to favor the adjustment of a suitable pessary or vaginal support, as represented in Fig. 7 on a former page.

Again, the patient now being in decubitus, the star (*e*) is taken as a *point d'appui* for the elevation of the uterus when anteverted or anteflexed to the lowest degree of pressure upon the bladder. The line of force, by graduated pressure, will be against the vesico-vaginal septum to arrow (1), and so on to arrow (*d*), thus forcing the uterus up to a fairly normal position without distressing the bladder, and at the same time making the adjustment of my vaginal support easy and effective, as is also shown by Fig. 7.

Whether either of these forms of columning of the vagina can be properly carried out with any other speculum than my own, it is not my purpose now to discuss. There is one point in the construction of my columns to which I would call particular attention, and that is the firmness and solidity given them in the lower third, and that is where the lateral blades of the instrument serve as a protection to the soft parts. There is no other speculum, with which I am familiar, possessing this advantage, and from this fact alone is to be found, I think, the explanation of the failure of many physicians, who use other instruments, to obtain that firmness of the column which is necessary to support and sustain the uterus at a mechanical disadvantage. The value of columning the vagina, however, as a principle of graduated pressure being recognized by the profession, the question as to the best mode of doing it will necessarily be settled by experience in practice. Whether I copied from Dr. Taliaferro or he from me, as shown by our respective publications, is of no consequence to the profession, but having been openly attacked by him in a way to disparage my labors regarding this subject, I feel it my duty to state some of the circumstances, which I think will fully justify my course from first to last, so far as he is concerned. This brings us to the third consideration of his claims to notice.

Third. *How he was probably first led to adopt the practice of packing the vagina.* In the spring of 1869, Dr. Montefiore J. Moses, having previously resided in Columbus, Georgia, for years on terms of great intimacy with Dr. Taliaferro, then resident in the same city, removed to New York. Very soon afterward he called upon me and became deeply interested in my special labors, at that time relating largely to vesico-vaginal fistule, its complications and its treatment with my new speculum and dilators in the knee-chest position, etc.; at the same time telling me how much his friend, Dr. T., was interested in these matters. In the course of the year he asked me to make out a list of my instruments, with which he had become familiar, saying that he wished it for Dr. Taliaferro. The list was furnished and placed in the hands of Messrs. Otto and Reynders. They manufactured the instruments, submitted them to my inspection and finally sent the case to Dr. T., at Columbus, Ga., April 18th, 1870. So far as I know these instruments gave entire satisfaction, if not to *all*, certainly to Dr. T., as I afterwards learned from his friend, Dr. Moses. So much for this assistance in putting him in the way of scientific study of diseases of women.

Again in 1877, just after my return from Europe, when I was engaged in treating a case of retroflexion of the uterus with fixation and prolapsed ovaries in St. Elizabeth's Hospital, in New York, by columning the vagina with dry cotton through my speculum, Dr. Moses accompanied me on one or two occasions to witness the procedure, and in expressing his satisfaction at the result, said that his friend, Dr. Taliaferro, was then residing in Atlanta, Georgia, and would be very much interested, he knew, in what he had seen of my method of treating such cases. How much Dr. Taliaferro was enlightened as to the general character of my mode of columning the vagina, at the time of which I am speaking, through the interest of his friend, I do not pretend to say. I simply state the fact, and know it was nearly a year prior to the publication of his first paper, in the spring of 1878.

Also, in the Autumn of 1881, about a year before the publication of Dr. Taliaferro's second paper, in which he makes the attack upon me, his pupil and associate, Dr. G. H. Noble, visited New York for the purpose of studying the diseases of women, and soon afterwards called upon me at my office to learn how he could best occupy his time while here. I gave him a note to my friend, Dr. Tanszky, in charge of the out-door clinic for diseases of women at Mount Sinai Hospital, of whom he could receive special instruction, and promised to show him all I could in my service at the Woman's Hospital. He expressed a particular desire to learn my mode of columning the vagina, and to see my instruments, for both of which he had ample opportunity at my office and in the hospital, as well as in the course of private instruction received of Dr. Tanszky.

On Dr. Noble's return to Atlanta, he becomes draughtsman to Dr. Taliaferro, and among his illustrations for Dr. T.'s paper is Fig. 9, copied on a former page, showing a modification of my column, which he had learned in my service at the Woman's Hospital.

But perhaps the most unqualified appropriation of Dr. T. without acknowledgment, is that of my perineal elevator, set upon the handle of Professor Simon's speculum, under the following pretext:

"The blade is flat and thin, like that of Nott's speculum, and flanged at the proximal end to separate and hold apart the nates. The handle terminates in a curve to fit the hand, and is light and more convenient for tamponing than an ordinary Sims' speculum." I reproduce the instrument here in two forms. Fig. 10 is the original, copied from Dr. Hamilton's work on surgery, 1873, and Fig. 11 the duplicate. No one need be told, I think, that the two thin and flat blades shown are of one and the same pattern. Dr. G. H. Noble, Dr. T.'s associate, saw the original instrument in my office, and saw me use it in connection with my speculum in the Woman's Hospital, seven or eight months ago, and probably took it home with him as a pattern for the modification here shown.

Fig. 10.

Fig. 11.

So much for some of the sources of Dr. Taliaferro's information and the manner in which he has profited by them in his two publications. His attempt to keep them out of sight under the guise of originality and of liberal acknowldgment of indebtedness, on almost every page of his first paper, to Dr. Sims for the precedence of suggestion, when he had no more to do with the development of graduated pressure as a system of treatment in the diseases of the vagina, uterus and ovaries, than Dr. T. had, is too patent to require here more than this passing notice. His mode of packing or cramming the vagina with sheep's wool, as shown by Fig. 8, copied from his first publication, I regarded from the first as not only unscientific and absurd, but dangerous and contrary to an intelligent understanding of the anatomy, physiology and mechanics of the pelvic organs. This, in connection with some of the circumstances just related, is the explanation of my taking no notice of his practice in my paper of 1878. The result of the abandonment of his first teaching after the publication of my paper, and his effort now to appropriate my flattened column of cotton and perineal elevator, as shown by the feeble attempt at modification, fully justifies, I think, my estimate of his pretensions from first to last.

In September, 1879, just one year after I submitted my paper to the American Gynæcological Society upon the "Mechanism of Uterine and Ovarian Displacements," etc., and six months after Dr. Tanszky's paper on "The Tamponade of the Vagina" appeared in the *New York Hospital Gazette*, Dr. Paul F. Munde read a paper before the same society, at its annual meeting held in Baltimore, entitled "Prolapse of the Ovaries." He stated that his attention had been first directed to the subject by a discussion at a meeting of the New York Obstetrical Society upon this topic, November 5th, 1873, nearly two months after the presentation of my paper as above mentioned. He said his record of 145 cases, collated from 1600 unselected gynæcological cases, showed that the affection was of frequent occurrence, and that the subject was one which had received but little attention from the profession, especially in this country, and in the plan of treatment he recommended a variety of modifications of Hodge's pessary.

As to the use of cotton for the same purpose, which he fully endorsed when the pessary could not be borne, he said: "This method of packing the vagina was first recommended by Taliaferro, of Georgia, for cases of cellulitis, metritis and oophoritis, and displacements in which a pessary cannot be borne, but Dr. Bozeman, I am informed, claims the priority of the principle." In his book, published more than a year later, entitled "Minor Surgical Gynæcology," 1880, he refers, under the caption of "Tamponade of the Vagina," to me and my claims with regard to the use of cotton in about the same words, and misapplies my designating term knee-chest for genu-cephalic, or knee-head position, and proceeds to describe the method of Dr. Taliaferro in accordance with the plan illustrated by Fig. 8, using, however, instead of dry wool, glycerated cotton, which is equally objectionable, on account of its packing in a hard lump. It, therefore, calls for no further notice here.

It is, I know, quite the custom for American medical writers to decry the labors of home contributors to the common fund of our knowledge, and to praise, sometimes unduly, those of foreign workers. Dr. Munde proved no exception to the rule in his paper referred to, on "Prolapse of the Ovaries."

The fact that in his report to the Mt. Sinai Hospital, of the Out-door Department for the diseases of women for the year ending Dec. 1, 1878, less than a year before he read his paper on "Prolapse of the Ovaries," he recorded 475 cases, nearly one-third of the 1600 cases upon which his statistics were based, and that out of even this large number not one case of prolapsed ovary is mentioned, while Dr. Tanszky, holding another service in the same hospital, as has been shown, found and diagnosed 16 cases of prolapsed ovaries out of 371 cases, is rather striking. Not only was this true with regard to Dr. Tanszky's service, but he treated these sixteen cases by columning the vagina with cotton in the knee-elbow position, which at my suggestion, he had then been using in the institution for five or six years.

As to my paper presented to the American Gynæcological Society a year before Dr. Munde read his, I would say that prolapsed and fixed ovaries in connection with retroflexion of the uterus with fixation formed a prominent feature of my remarks. This was also true of the remarks of Dr. Tanzsky published in the *N. Y. Hospital Gazette*, as an endorsement of my views upon the subject, some six months, also, before Dr. M. read his paper. Yet, with this attention bestowed by both of us upon the subject of prolapsed ovaries, the Doctor did not find it desirable or convenient even to mention the fact of these prior observations!

As to Dr. Taliaferro's paper, however, in 1878, recommending the stuffing of "the entire vaginal canal from its vault to the floor of the pelvis, completely and compactly" with dry wool, Dr. M. acknowledged his deep sense of obligation, and adopted without question the teaching, including in the same line of instruction the treatment of oophritis, but, in adopting the teaching of Dr. T., he used glycerated cotton instead of dry wool. In Dr. T.'s paper referred to, there is not the slightest mention of oophoritis or prolapsus of the ovaries, much less, treatment of the same by dry wool packing of the vagina.

That Dr. Munde may possibly have observed sixteen hundred unselected gynæcological cases, out of which he found one hundred and forty-five cases of prolapsed ovaries, it is not my purpose to question. Considering, however, that this large number of cases was observed in a period of nine months, to-wit: from December 1, 1878, when he made his report to the Mt. Sinai Hospital, and had not recorded a single case, to September, 1879, when he read his paper before the American Gynæcological Society, we are naturally led to the conclusion that he must have encountered an unheard of epidemic of prolapsed ovaries at or just before the time he prepared his paper upon the subject.

Even in his book (1880) I find under the heading of "Tamponade of the Vagina," twenty pages devoted to the subject of using, instead of dry wool, glycerated cotton in the vagina, which he learned so well from Dr. Taliaferro, and yet not one line is given to a description of my method of columning the vagina with dry cotton, further than to adopt my designating word "column," as he does Dr. Tanszky's term "tamponade of the vagina," giving credit for neither.

The remarks contained in this paper justify, I think, the following conclusions:

1. That cicatricial contractions of the vagina (Kolpostenosis) and fixation of the uterus by pelvic exudations and adhesions following protracted labor, constitute the prime, and often insurmountable, obstacles to the cure of urinary and fecal fistules, and also of displacements of the uterus and ovaries.

2. That previous to the year 1855, notwithstanding the fact that unopposed or passive vaginal dilatation by the pubo-sacral or univalve-acting speculum in the knee-elbow and exaggerated knee-elbow positions had been, for nearly a quarter of a century, thoroughly understood in Europe and in this country, and some success had been reached in the closure of simple and small fistules, little or no attention had been given to the stretching treatment of vaginal contractions and pelvic adhesions as the real obstacles to vaginal dilatation and the restoring of uterine mobility, further than to divide simple cicatricial bridles as they happened to appear in the way of immediate exposure of coexisting fistules.

3. That, during the year above indicated, graduated vaginal and uterine pressure with pieces of sponge compressed in oil-silk bags of graded sizes in the form of vulvo-vaginal and intra-vaginal dilators, was first associated with immediate division of cicatricial bands as a systematic mode of gradual preparatory treatment; and that it was done with the idea, not only of overcoming such obstacles in and around the vagina as prevented exposure of the coexisting fistule, but of carrying vaginal dilatation and uterine elevation beyond the limits of cicatricial resiliency and fibro-pelvic restraint.

4. That with this forward step in the utilization of graduated pressure, together with the aid afforded to the closure of large fistulous openings by drawing down the uterus and fixing it with the button suture in the knee-elbow and knee-chest positions, unprecedented success was attained, with preservation of the functions of the organs involved, in an average proportion of complicated cases, including retroflexion of the uterus with fixation and with displacement of the ovaries.

5. That the pubo-sacral or univalve-acting speculum, with assistant always to hold it, while adapted, in simple cases, elevating and supporting the perineo-rectal wall in all the anterior positions of the patient, it failed in a large proportion of cases with or without relaxed vaginal walls, because it exerted no controlling influence over the anterior wall of the vagina in its normal condition, and but little over it and the lateral walls when they were the seat of outstretched cicatricial bands. For the latter reason the instrument did not favor the highest aims of graduated vaginal and uterine pressure in the procedure of gradual preparatory treatment with incisions, but rendered kolpokleisis and kolpoplecosis necessary expedients for the relief of urinary and local fistules.

6. That the intra-ischial or bilateral-acting speculum, self-acting and self-sustaining, requiring no assistant to hold it (1867), was found not only to dilate the vulva to the fullest extent and give steadiness to all the walls of the vagina, but to develop, hitherto concealed, cicatricial contractions and far-reaching, flattened, inodular masses, which with the univalve speculum had before passed unnoticed. For these reasons the highest limit of success, with this new principle of dilatation, was attained through graduated vaginal and uterine pressure as preparatory treatment, which is essential to absolute cure of urinary and fecal fistules and the avoidance of kolpokleisis and kolpoplecosis.

7. That with the intra-ischial or bilateral-acting speculum, columning the vagina with dry cotton for the relief of prolapsus, and ante- and retro-displacements of the uterus, simple or complicated with adhesions and prolapse of the ovaries, was the natural outgrowth of columning the same organs with sponges in oil-silk bags, as pointed out in connection with cicatricial contractions, and that the system as now employed can only be regarded as a modification or extension of the cotton under another form of graduated pressure.

By graduated pressure thus made to the walls of the vagina and to the uterus and its appendages, the large class of cases indicated can be treated on rational and scientific principles, and it is now possible to reduce to very exceptional cases the necessity of bloody operations for superficial lacerations of the cervix uteri, or for prolapse of the anterior and posterior walls of the vagina.

8. That, however successful columning the vagina may be in relieving the class of cases indicated, it is still necessary in a large proportion of them to maintain the cure for a time by strengthening the counteracting forces residing in the vaginal walls and uterine ligaments with some mechanical appliance introduced into the vagina, either manageable or not by the patient. In short, the support of the uterus and ovaries in an elevated position, with elongation of the walls of the vagina, requires, under such circumstances, Hodge's pessary or the elastic vaginal support before described (1878.) When the preparatory treatment is properly carried out, this latter support fulfills all the indications better than any other instrument hitherto devised, it being entirely managed by the patient herself.

9. That distortions of the vagina due to prolapsus and ante- and retro-displacements of the uterus, associated or not with prolapse of the ovaries, as results of endometritis, metritis or peri-metritis, or all three together, coupled with plastic exudations and adhesions, cannot be overcome by cutting operations upon the infra-vaginal portion of the cervix uteri, or either, or both of the walls of the vagina, or all three structures together, as first insisted upon in 1878, and that such operations have no surgical importance in the mechanics of the pelvic organs; the distortions of the vagina remaining the same after their performance as before.

10. That lacerations of the cervix uteri within the last few years, as factors concerned in the causation of neurological complications and malignant developments, have been greatly overestimated, and that the most of the schemative illustrations of these so-called lesions to be found in the gynæcological literature of the day, whether transversely, unilateral or bilateral, or transversely and antero-posteriorly trilateral or quadrilateral, are overdrawn and have no foundation in a true study of uterine pathology.

11. That the recognition of the frequent existence of prolapse of the ovaries in relationship with ante- and retro-displacements of the uterus with and without fixation, was a most important step as regards scientific treatment (1874), and that it is now only by a clear understanding of this relationship of the parts in such abnormal conditions, further advances are to be made in the line of successful practice.

12. That the disposition of writers to misapply the designating term knee-chest position for the exaggerated knee-elbow, genu-cephalic or knee head position is unwarranted, and opposed to true scientific progress in the treatment of an important class of cases which are the most difficult to manage and the least understood by the profession at large.

## Figures and Tables

**Fig. 5. f1:**
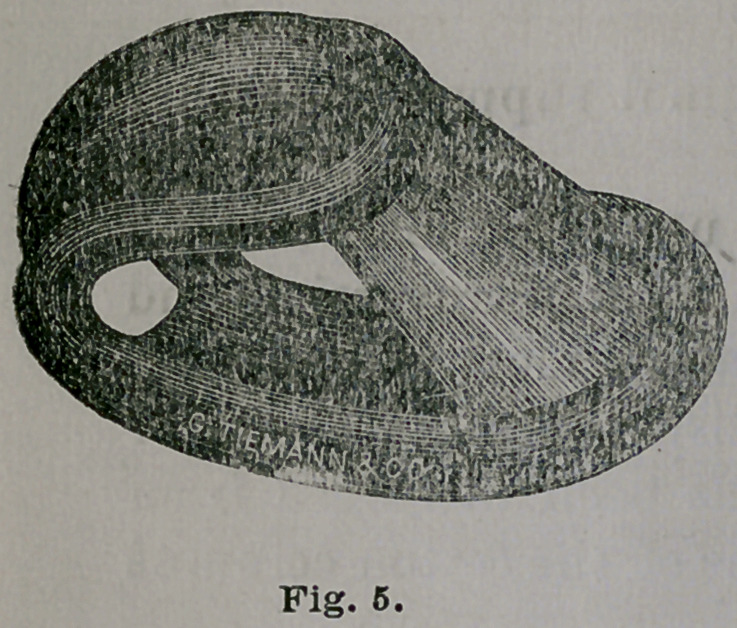


**Fig. 6. f2:**
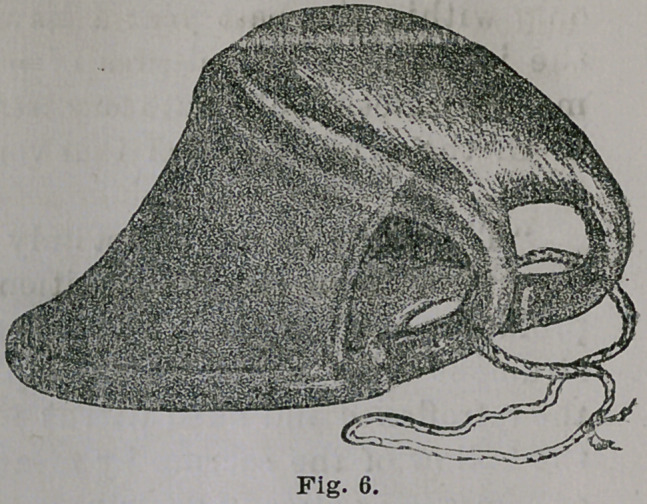


**Fig. 7. f3:**
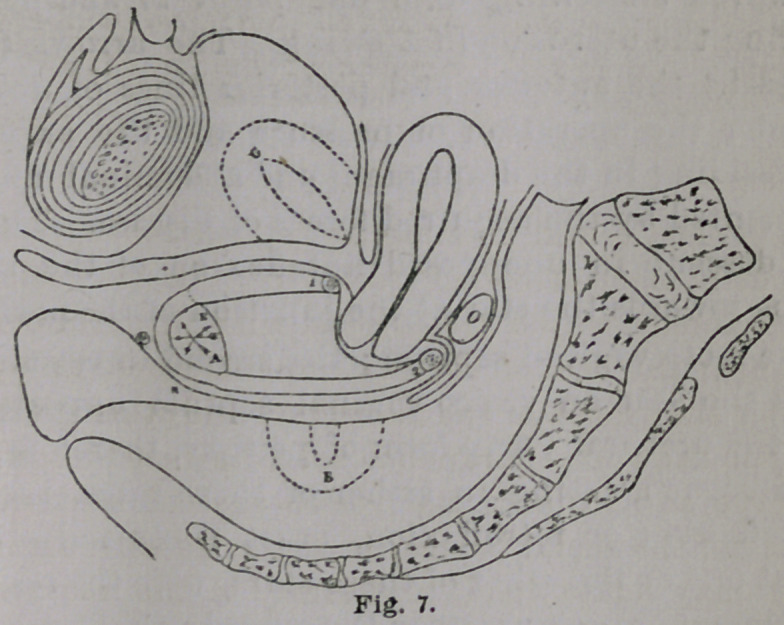


**Fig. 8. f4:**
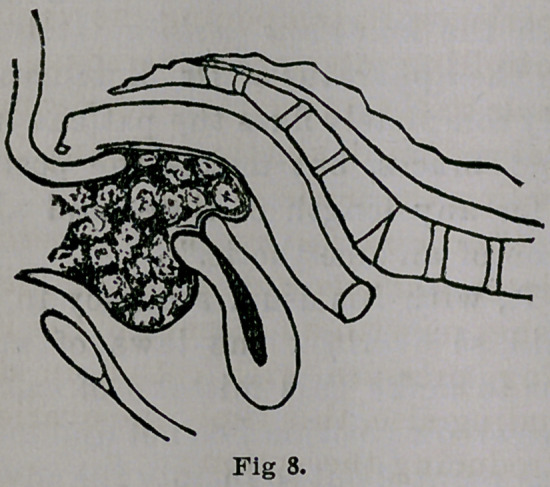


**Fig. 9. f5:**
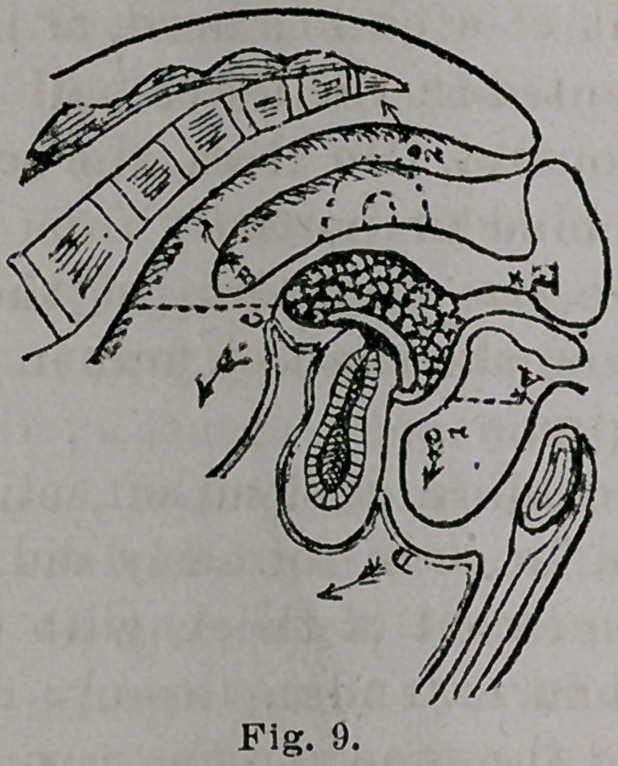


**Fig. 10. f6:**
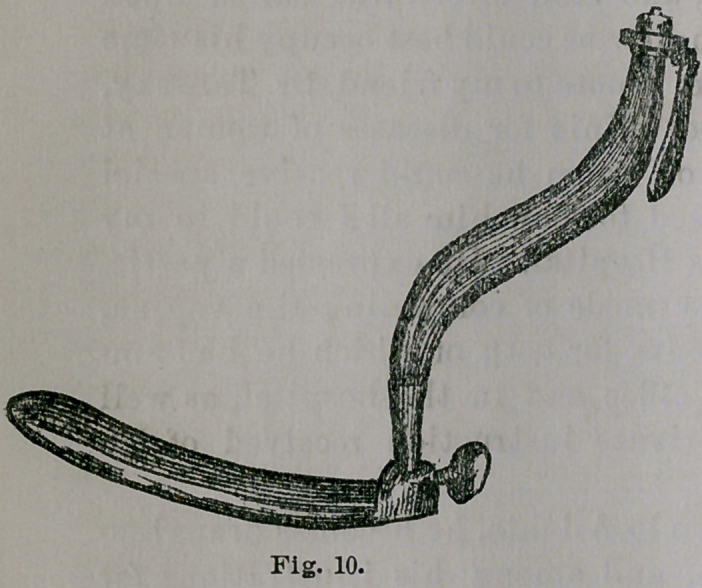


**Fig. 11. f7:**